# A case of hepatic sarcomatoid cholangiocarcinoma with diaphragmatic and right lower lobe lung invasion

**DOI:** 10.1093/jscr/rjaf047

**Published:** 2025-02-10

**Authors:** Tsung Chiao Tsai, Che Min Su

**Affiliations:** General Surgical Department, National Cheng Kung University Hospital, No. 138, Shengli Rd., North Dist., Tainan City 704, Taiwan (R.O.C.); General Surgical Department, National Cheng Kung University Hospital, No. 138, Shengli Rd., North Dist., Tainan City 704, Taiwan (R.O.C.)

**Keywords:** cholangiocarcinoma, immunohistochemical stain, hepatocellular carcinoma (HCC), sarcomatoid intrahepatic cholangiocarcinoma

## Abstract

Hepatic sarcomatoid cholangiocarcinoma is a rare, accounting for <1% of cases and aggressive malignant neoplasm. This report presents a case of a 35-year-old female patient who presented with right shoulder pain, general weakness, anorexia, and weight loss. Imaging studies revealed a hepatic mass in segment S8, with invasion into the diaphragm and right lower lobe of the lung. The patient underwent surgical resection, and the pathological diagnosis confirmed hepatic sarcomatoid cholangiocarcinoma. This case report discusses the clinical presentation, diagnosis, and treatment of this rare malignancy, emphasizing the importance of early diagnosis and aggressive management.

## Introduction

Hepatic sarcomatoid cholangiocarcinoma is a rare and highly malignant liver tumor. Its clinical presentation is variable, making early diagnosis challenging. The prognosis is poor [1–4]. This report describes a typical case to increase awareness of this disease and to explore its diagnostic and therapeutic strategies.

## The case

**Figure 1:**
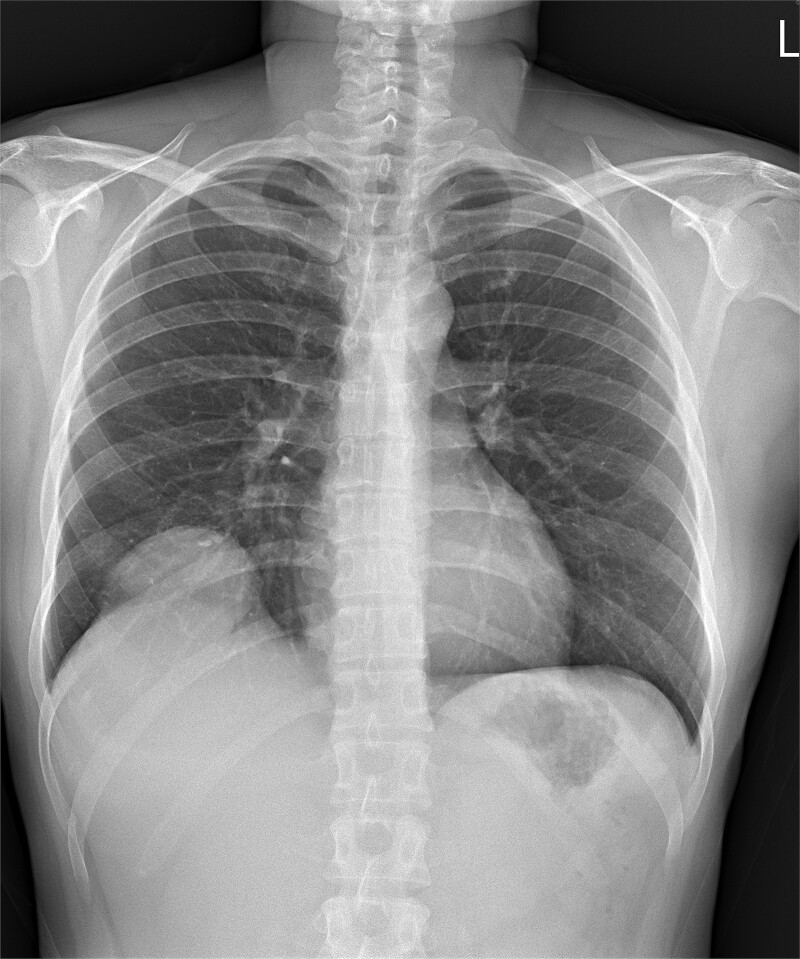
Chest X ray image of sarcomatoid cholangiocarcinoma with direct invasion into the diaphragm and lung.

A 35-year-old woman with a history of prior alcohol use and tobacco smoking presented with a 2–3 month history of right shoulder pain, followed by generalized weakness, poor appetite, and abdominal discomfort. She also reported right rib pain, unintentional weight loss (3 kg within 3 months), and night sweats. She denied fever, upper respiratory infection symptoms, chest pain, shortness of breath, abdominal pain, dysuria, or diarrhea. Initial investigations at another hospital, including a chest X ray (Fig. 1), abdominal computed tomography (CT) scan, revealed a hepatic mass at segment 8 (S8). Tumor markers, hepatitis B virus, and hepatitis C virus serologies were negative.

**Figure 2:**
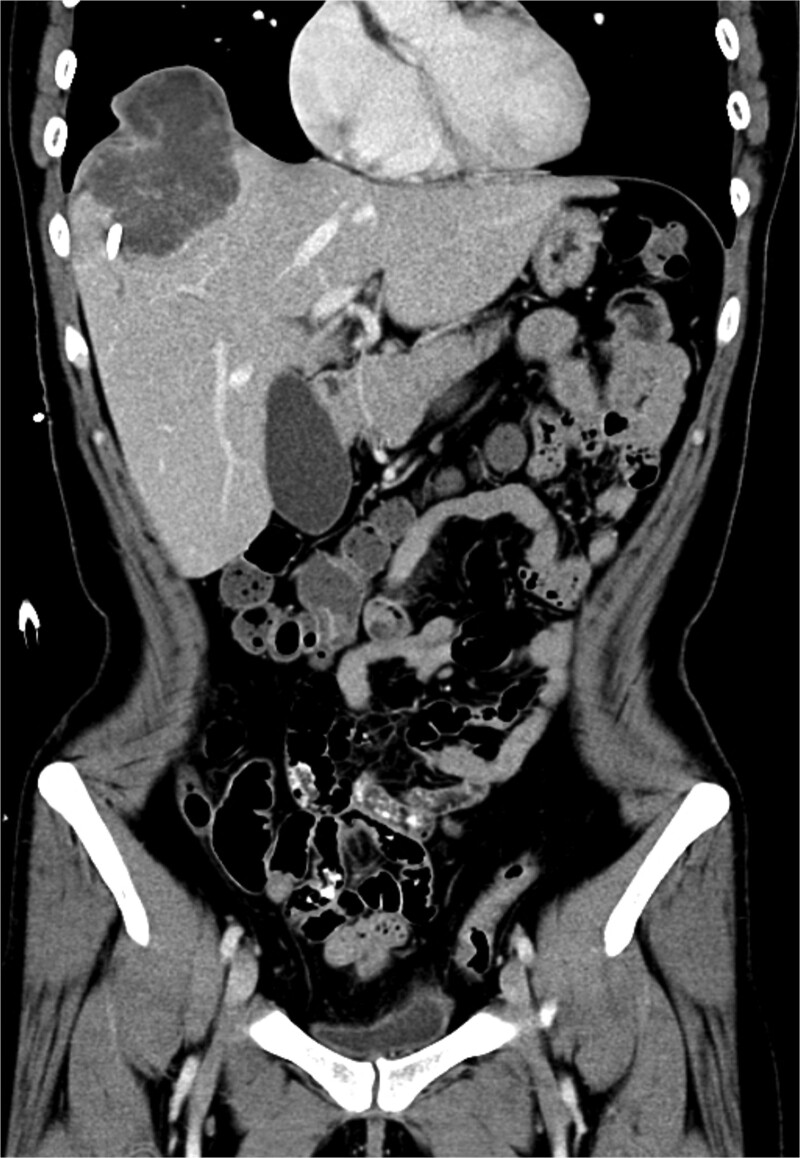
Coronal view of a CT image of sarcomatoid cholangiocarcinoma.

**Figure 3:**
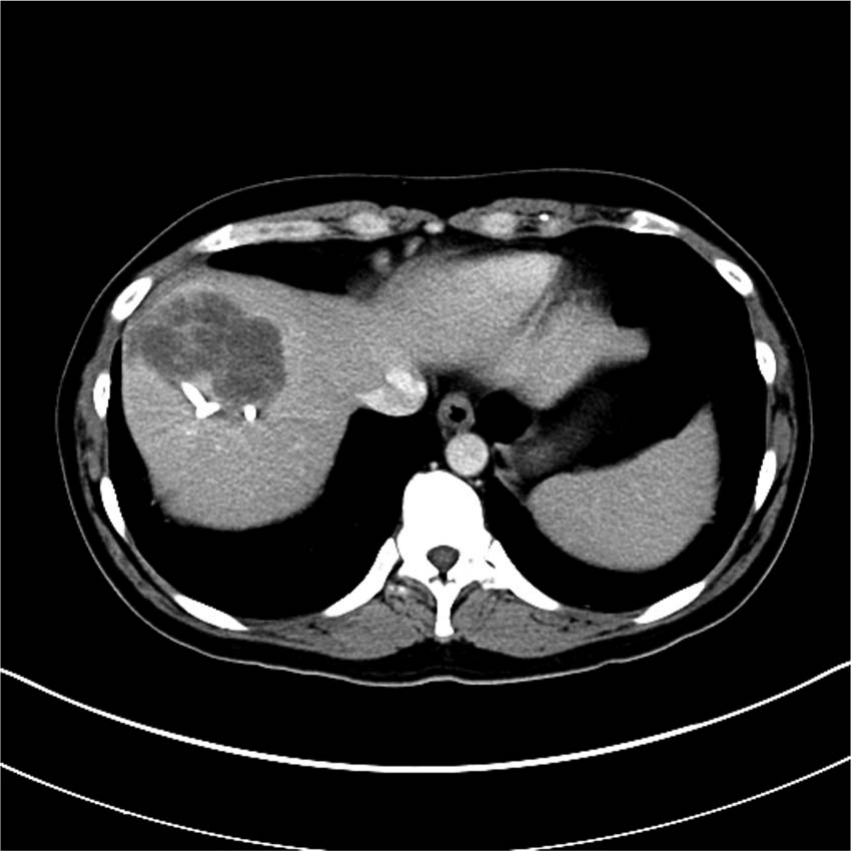
Axial view of CT image of abdomen.

Upon admission to the GI ward, a repeat abdominal CT scan suggested a possible liver abscess. Drainage was performed on September, 2024, and empirical antibiotic therapy with ceftriaxone and metronidazole was initiated. Despite initial drainage and antibiotics, the patient continued to experience intermittent fever. A subsequent CT scan revealed a heterogeneous lesion measuring ~7.5 × 8.5 cm at S8 (Figs 2 and 3). A sono-guided liver biopsy was performed, and the pathological report indicated hepatic sarcomatoid carcinoma with variable positivity for CK and CK7, and negativity for CK20, glypican-3, ERG, and CD34 stains, suggesting a possible cholangiocarcinoma component. A general surgery (GS) consultation was obtained for suspected liver tumor management.

At the GS OPD, surgical intervention was discussed, and the patient consented to proceed with a laparoscopic S8 segmentectomy. She was readmitted for surgery under the diagnosis of hepatic sarcomatoid carcinoma at S8.

### Surgical procedure and postoperative course

Then, the patient underwent a planned laparoscopic S8 segmentectomy. Intraoperatively, the tumor was found to have directly invaded the diaphragm and right lower lobe of the lung, necessitating conversion to an open approach via an upper midline incision. The procedure was then completed as an open liver S8 segmentectomy and right lower lobe (RLL) wedge resection.

### Intraoperative findings

An 8.0 × 8.0 cm sarcomatoid carcinoma of the liver at S8 with direct invasion into the diaphragm and RLL was identified.

### Surgical technique

**Figure 4:**
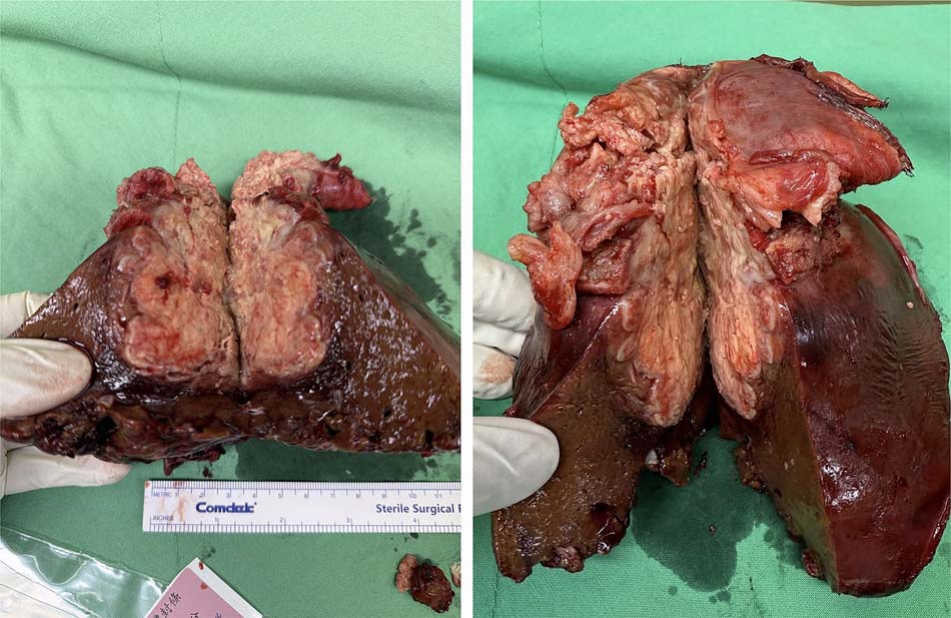
Specimen of sarcomatoid cholangiocarcinoma.

Laparoscopic S8 monosegment liver resection was initiated, including S8 pedicle resection and parenchymal division using an EndoGIA stapler and harmonic scalpel. Due to tumor invasion, the procedure was converted to open. Parenchymal dissection was completed with the harmonic scalpel. Pringle maneuver was employed with total clamping time of 78 minutes. Diaphragm resection and RLL wedge resection were performed (Fig. 4). The diaphragm was repaired with silk sutures. Specimen margins were confirmed to be free of tumor, although tumor rupture at the medial site occurred due to severe adhesion to the diaphragm and RLL. Cholecystectomy and hilum lymph node dissection were also performed. A Jackson-Pratt drain was placed over the cutting surface. A chest tube was placed over right side.

Postoperatively, the patient was transferred to the Surgical Intensive Care Unit (SICU) and subsequently to the regular ward on postoperative day (POD) 1. Postoperative anemia required blood transfusion. Follow-up chest X-rays showed gradual improvement of RLL radioopacity, allowing for chest tube removal. Wound healing was satisfactory, with localized fat necrosis that resolved with daily dressing changes. Genetic mutation analysis was performed as recommended by the Department of Medical Genetics. The final pathology report confirmed sarcomatoid cholangiocarcinoma, poorly differentiated. Margin reported all Uninvolved by invasive carcinoma. The patient was discharged in stable condition on POD 16.

## Discussion

This case presents a rare and challenging instance of hepatic sarcomatoid cholangiocarcinoma with direct extension into the diaphragm and right lower lobe of the lung. Sarcomatoid cholangiocarcinoma is a rare variant of cholangiocarcinoma, characterized by a biphasic histology consisting of malignant epithelial cells and a spindle cell component resembling sarcoma [5]. This histological heterogeneity contributes to its aggressive behavior and poor prognosis. The incidence of sarcomatoid cholangiocarcinoma is low, representing only a small percentage of all cholangiocarcinomas. Its rarity often leads to diagnostic challenges, as the initial presentation can mimic other hepatic lesions, such as hepatocellular carcinoma, metastatic tumors, or even benign conditions like liver abscess, as observed in this case.

The direct invasion of the tumor into the diaphragm and lung is a particularly aggressive feature of this case. This extensive local spread necessitated a conversion from the planned laparoscopic approach to an open procedure. The ability to adapt the surgical strategy intraoperatively was crucial for achieving complete tumor resection. Although clear margins were achieved, the intraoperative rupture of the tumor due to severe adhesions raises concerns about potential microscopic spread and underscores the need for close postoperative surveillance and consideration of adjuvant therapies.

Surgical resection remains the mainstay of treatment for localized cholangiocarcinoma, including sarcomatoid variants. However, the prognosis for patients with advanced disease or extensive local invasion, as seen in this case, remains poor [2]. The role of adjuvant therapies, such as chemotherapy or radiation therapy, in improving outcomes for these patients is still under investigation. Given the rarity of this condition, there is limited evidence to guide optimal treatment strategies. Further research, including larger case series and clinical trials, is needed to better understand the natural history of sarcomatoid cholangiocarcinoma and to develop effective therapeutic interventions. Genetic mutation analysis, as performed in this case, may provide valuable insights into the molecular characteristics of the tumor and potentially identify targets for personalized therapies in the future.

## Conclusion

Hepatic sarcomatoid cholangiocarcinoma is a challenging disease. This case demonstrates the successful management of a rare presentation of hepatic sarcomatoid cholangiocarcinoma with direct invasion into the diaphragm and lung through a combined laparoscopic and open surgical approach. It emphasizes the importance of a high index of suspicion, meticulous pathological evaluation, and adaptable surgical strategies in managing this rare and aggressive malignancy. Further research is warranted to improve outcomes for patients with this challenging disease.
